# Vaccination against Hepatitis B among health care workers in the Bamenda Health District: influence of knowledge and attitudes, Cameroon

**DOI:** 10.11604/pamj.2021.40.216.16856

**Published:** 2021-12-09

**Authors:** Anye Muriel Ngum, Sobngwi Joëlle Laure, Xavier Tchetnya, Tabe Armstrong Tambe, Claude Nkfusai Ngwayu, Frankline Sevidzem Wirsiy, Catherine Atuhaire, Samuel Nambile Cumber

**Affiliations:** 1 School of Health Sciences, Catholic University of Central Africa, Yaoundé, Cameroon,; 2 Department of Microbiology and Parasitology, Faculty of Science, University of Buea, Buea, Cameroon,; 3 Cameroon Christian University Bali, Institute of Health and Biomedical Science Bali, Bali, Cameroon,; 4 Department of Public Health, School of Nursing and Public Health, University of Kwa-Zulu Natal, Durban, South Africa,; 5 Pfizer scholar, One Young World (OYW), 10 Queen Street Place, London, United Kingdom,; 6 Faculty of Medicine, Department of Nursing, Mbarara University of Science and Technology, Mbarara, Uganda,; 7 Institute of Health and Care Sciences, The Sahlgrenska Academy at the University of Gothenburg, Gothenburg, Sweden,; 8 Office of the Dean, Faculty of Health Sciences, University of the Free State, Bloemfontein, South Africa

**Keywords:** Hepatitis, vaccination, knowledge, attitudes, health care workers, Bamenda Health District, Cameroon

## Abstract

**Introduction:**

Hepatitis B virus (HBV) infection is one of the most serious occupational hazards faced by healthcare workers (HCW). This study aimed at assessing the influence of knowledge and attitudes of HCWs in the Bamenda Health District (BHD) on their vaccination status.

**Methods:**

this was a cross-sectional analytic study carried out in Bamenda health district, Cameroon. Random sampling method was used to select 10 private, 10 public, and 4 confessional health facilities, from which 280 HCW were included in the study by convenience sampling. Data were analysed using Epi Info 7 and presented using tables, figures, and percentages.

**Results:**

the vaccination coverage among HCW in the BHD was found to be 13.9%. Healthcare workers who had no knowledge of the minimum number of doses for complete primary HBV vaccination were less likely to be vaccinated than those who had knowledge (p = 0.00). Healthcare workers who had been tested for HBsAg were more likely to be vaccinated than those who had not done the test (p = 0.00). Among HCW (90.7%) who knew they were more at risk of contracting HBV, 98.6% knew it can be prevented out of which 72.6% reported that vaccination is the most effective means of prevention; only 13.9% of HCW were vaccinated. Other factors could have influenced the vaccination status of HCW; high cost of the vaccine, lack of time for vaccination, negligence, and the non-availability of the vaccine.

**Conclusion:**

awareness should be created among HCW and they should be encouraged to go for HBsAg screening and those who are negative should receive a full dose of HBV vaccine. Also, the vaccine should be subsidized and made available to all HCW in the BHD.

## Introduction

Hepatitis B is a potentially life-threatening liver infection caused by the hepatitis B virus (HBV) and is a major global health problem [1]. HBV is more infectious than the other blood-borne pathogens, with a 30% risk of contamination after exposure by a single needle stick injury compared to 3% for HCV and 0.3% for HIV [2]. Hepatitis B virus is also transmitted by exposure to infected blood or body fluids such as semen and vaginal fluids, and prenatally from mother to child [3]. It is estimated that more than two billion people worldwide have been infected by HBV at one point in their lives and about 350 million of them suffer from chronic HBV infection. Hepatitis B infection is one of the most serious and prevalent health problems, affecting more than 2 billion people worldwide with an estimated global mortality of 0.5 to 1.2 million deaths per year [4-6]. Areas with a low prevalence include North America, Western and Northern Europe, Australia, and parts of South America and the carrier rate here is less than 2%, and less than 20% of the population is infected with HBV [7-9].

Cameroon falls in the group of countries having a prevalence of >8%. In 1993 the prevalence of HBV was 6.7% [10]; while in 2001 a prevalence of 10.7% was recorded in Cameroon [11]. Eleven years later the prevalence of Hepatitis B in Cameroon was 12.14% [12]. Due to the apparent lack of knowledge about hepatitis B, most governments have seriously not considered hepatitis B prevention as a topmost priority in health care. However, in Cameroon knowledge about hepatitis is still very low. Unfortunately, there is little data on Cameroon concerning knowledge of HBV infections among its HCWs. A study carried out on 395 HCW showed that they had sufficient knowledge and their vaccination coverage was 69% [13]. Also, a study carried out in India on 540 dental health care workers showed that they knew [14]. Nevertheless, some studies have been carried out and there was insufficient knowledge among health personnel; a study carried out in South West Cameroon with a sample size of 237 HCWs [15] with vaccination coverage of 5.5%, a study carried out on 343 HCWs and they concluded that knowledge is associated to vaccination status of HCWs. This study aimed at assessing the influence of knowledge and attitudes of HCWs in the Bamenda Health District (BHD) on their vaccination status.

## Methods

**Study design, settings and population:** an analytic cross sectional design was used in this study to assess the influence of the knowledge and attitudes of HCWs on their vaccination status. This method was chosen due its ability to provide appropriate and relevant data). A Random sampling method was used to select 24 (4 confessionals, 10 public, and 10 private) out of 35 HF. A non-probabilistic method of sampling (sampling by convenience) was used to select 280 HCW within the 24 HF to participate in the study. The study was carried out at the BHD. It is one of the 19 health districts in the North West (NW) region located at the heart of the North West Regional Head Quarter with a population of about 341,864 inhabitants (2010 statistics). It is bounded to the north by Bafut, to the south by Santa, to the west by Bali, and the east by Tubah Health Districts. The BHD is comprised of 17 Health Areas (HA) and 35 health facilities (HF). It is made up of confessionals, public and private HF. However, many other private HF are operating in anonymity (not cooperating with the BHD) while others are registered at the district health service but are not operational.

**Sample size:** the sample size was calculated using the Lorenz formula:


$$n=\frac{{\left(t\right)}^{2*}p(1-p)}{{\left(e\right)}^{2}}$$

Where: p = vaccination against HBV coverage among surgical residents in Yaoundé [16]; e = standard error; n = required population size; t = confidence level at 95%.


$$n=\frac{{\left(1.96\right)}^{2*}0.245(1-0.245)}{{\left(0.05\right)}^{2}}=284.24$$

### Selection criteria

**Inclusion criteria:** healthcare workers in the Bamenda health District that were willing to provide consent were included.

**Exclusion criteria:** students who were on internship in the BHD and part-time HCW were excluded.

**Sampling technique:** the BHD covers three subdivisions; HCW were recruited from all 3 subdivisions (sub-divisions 1, 2, and 3). A Random sampling method was used to select 24 (4 confessionals, 10 public, and 10 private) out of 35 HF. A non-probabilistic method of sampling (sampling by convenience) was used to select 280 HCW within the 24 HF to participate in the study. This means that HCW who were available during the time of study and were willing to participate were included in the study. All HCW were given equal chances to participate in the study; we had to pass through each HF more than once depending on the number of shifts in the HF. Though Alabukam and Ntankah (HF found in the BHD) were selected during random selection were not included in the study because they were not operational during the period of the study. In this study, all workers involved in the care and treatment of patients were involved irrespective of their age, sex, or qualification.

**Data collection:** a pre-test was done on five health care workers in the Mbengwi Health District to ensure that the questions were clear and easy to understand after which corrections were made before it was administered to HCW in the BHD. The questionnaires (for the number of HCW who were present and had given their consent) were handed to the chief or head of each unit and the instructions were explained to them. The sample populations were briefed on the reasons for carrying out the research and assurance to maximum confidentiality given to all the participants. Later a total of 280 questionnaires were distributed to the HCW who accepted to participate and were answered and collected on the spot. Data was collected using a structured questionnaire, divided into five sections, identification of HF, socio-demographic characteristics of HCW, knowledge, attitudes of HCW regarding HBV, and vaccination against HBV status of HCW. These questionnaires were self -administered. This study lasted for 12 months (January 2014 to January 2015).

**Ethical considerations:** ethical clearance was obtained from “Comite d´Ethique Institutionnel de la Recherche pour la Sante Humaine” (CEIRSH); meanwhile, participants´ consent was sought to maintain a high level of confidentiality before inclusion in the study. Authorisations were obtained from the Regional Delegation for Public health North West region, BHD, and from all the 22 health facilities in which the study was carried out.

**Data analysis:** data were entered and analysed using Epi info 7. Descriptive analysis was done specifically using frequency tables and graphs with a calculation of means (for quantitative variables like age), and standard deviation where necessary, as well as proportion (for qualitative) for specific independent variables. Bivariate and multivariate analysis was done. Chi-square, ANOVA, and Fischer exact test (used when modality <5) were used to determine the association between dependent and independent variables. Those that were significant (p-value< 0.05) were put in the logistic regression model together with possible confounders (age, sex, marital status).

## Results

**Demographic data:** of the 280 participants enrolled 180 (64.29%) were females while 100 (35.71%) were males giving us a male: female ratio of 1: 1.8. Looking at marital status, 124 (44.44%) of HCW were single or widowed and 155 (55.56%) were married. Analysing for a level of education, 179 (63.93%) had tertiary education, 87 (31.07%) had secondary education and 14 (45.00%) had primary education. In the tertiary level of education, 100 (68.03%) of HCW were from the private sector as compared to 79 (58.96%) as seen in Table 1.

**Table 1:** frequency and percentage distribution of respondents according to gender

Variables	Group	Public	Private	Total
		n (%)	n(%)	n(%)
Location of HF (n=280)	Urban	106 (79.70)	135 (91.84)	241 (86.07)
	Rural	27 (20.30)	12 (8.16)	39 (13.93)
Sex (n = 280)	Male	38 (28.57)	62 (42.18)	100 (35.71)
	Female	95 (71.43)	85 (57.82)	180 (64.29)
Marital status (n = 279)	Single/Widow	38 (28.57)	86 (58.90 )	124 (44.44)
	Married	95 (71.43)	60 (41.10)	155 (55.56)
Level of education (n=280)	Primary	8 (6.02)	6 (4.08)	14 (5.00)
	Secondary	46 (34.59)	41 (27.89)	87 (31.07)
	Tertiary	79 (59.40)	100 (68.03)	179 (63.93)

**Frequency and percentage distribution of respondents according to gender qualification:** out of 280 respondents, nurses were 99 (35.4%), laboratory technicians 60 (21.2%), nursing assistant 46 (16.5%), nurse specialist 30 (10.7%), specialist were 11 (3.9%), general practitioner were 11 (3.9%), assistant laboratory technicians were 10 (3.6%), pharmacists were 8 (2.9%) and finally radiologists 5 (1.8%).

**Figure 1:**
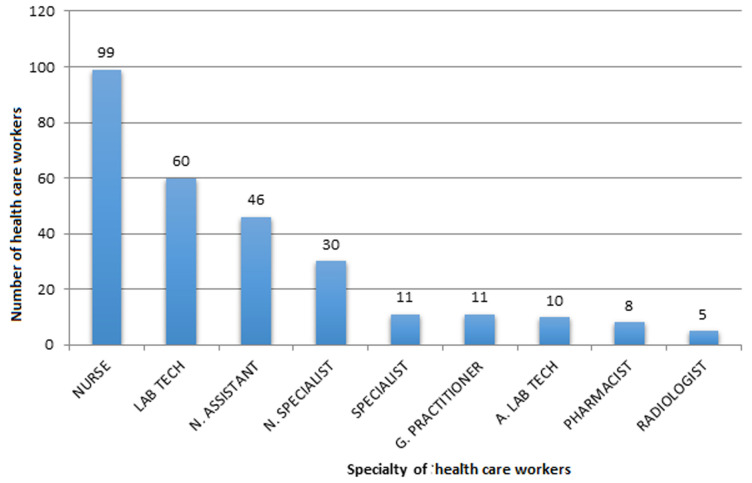
distribution of respondents according to specialty in the BHD

**Distribution of respondents by specialty:** Figure 1 illustrates the different specialties represented in the study. Out of the 280 respondents, nurses were 99 (35.4%), laboratory technicians 60 (21.2%), nursing assistant 46 (16.5%), nurse specialist 30 (10.7%), specialist were 11 (3.9%), general practitioner were 11 (3.9%), assistant laboratory technicians were 10 (3.6%), pharmacists were 8 (2.9%) and radiologists 5 (1.8%).

**Vaccination coverage among HCW:** out of the 280 respondents, 39 (13.9%) had been correctly vaccinated. Those who were either not vaccinated or incorrectly vaccinated reported 241 (86.1%).

**Figure 2:**
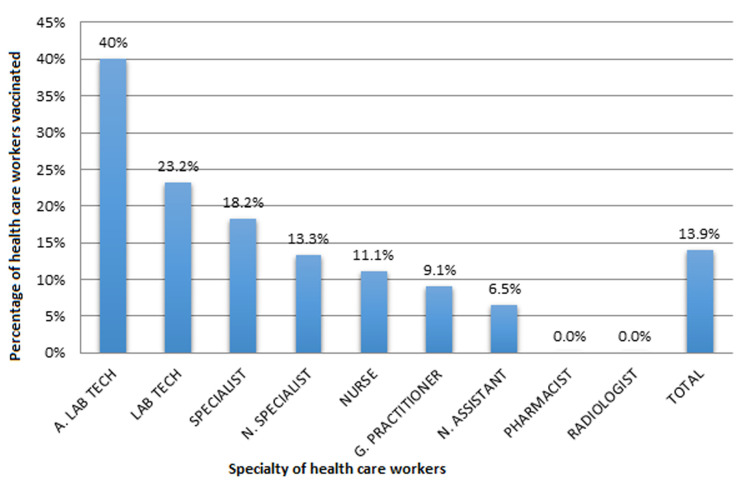
distribution of respondents according to vaccination coverage of HCW in the BHD by specialty

**Vaccination coverage by specialty:** the vaccination coverage was not the same for all specialties. The vaccination coverage was 40% (4/10) for assistant laboratory technicians while none of the pharmacists and radiologists recruited in this study were vaccinated as seen in Figure 2.

**Figure 3:**
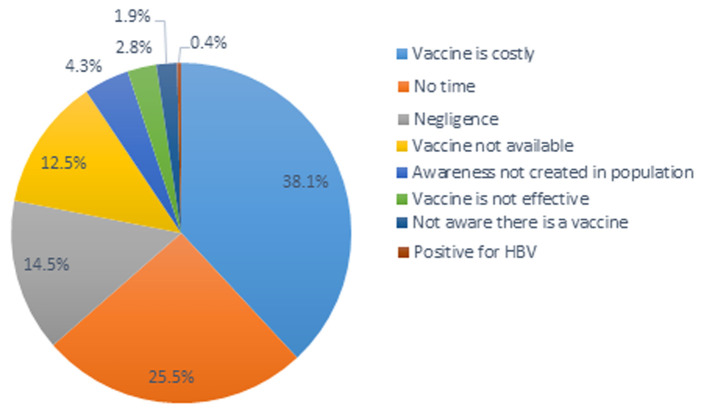
distribution of respondents according to reasons for non-vaccination among HCW

**Reasons why HCW were not vaccinated:** the most reported reasons for non-vaccination among HCW were 34.6% (97/255) for the vaccine is costly, and 23.2% (65/255) for lack of time while the least response was from those who were already infected with the virus 0.4% (1/255) as shown in Figure 3.

**Association between socio demographic factors and vaccination status:** the relationship between longevity in service and vaccination status of HCWs was significant (p=0.03). The relationship between age, sex, marital status, level of education, location of HF, specialty of HCW and vaccination status was not significant (p>0.05).

**Association between Knowledge and vaccination status of HCW:** knowledge on healthy skin and infected blood, knowledge on minimum number of vaccine dose was significantly associated to the vaccination status of HCWs (p<0.05). Knowledge regarding other aspects of HB was not statistically related to vaccination status of HCWs.

**Association between attitudes and vaccination status of HCW:** attitudes of HCWs regarding safety of vaccine and screening for HBsAg were significantly related to the vaccination status of HCWs (p< 0.05). Attitudes regarding all other aspects of HB were not significantly related to the vaccination status of HCWs (p>0.05).

**Independent variables that could influence the vaccination status of HCW:** all variables from bivariate analysis with a p value < 0.05 (longevity in service, knowledge on healthy skin and infected blood as a risk factor, knowledge on minimum number of doses for complete primary HB vaccination, recommendation of HCW for a HB patient, attitudes regarding safety of vaccine and screening for HBsAg) and possible confounding factors (age, sex, marital status) were put into logistic regression model. HCW who had no knowledge on the minimum number of dose for a complete primary HB vaccination were less likely to be vaccinated than HCW with knowledge [AOR = 0.25; 90% CI: 0.10-0.62, p = 0.00] as shown in Table 2. Also, HCW who have done the HBsAg test were more likely to be vaccinated than those who had not done the test [AOR = 4.09; 90% CI: 1.56-10.68, p=0.00].

**Table 2:** independent variables that could influence the vaccination status of HCW

Variable	Category	Adjusted Odd ratios (AOR)	95% CI	p-value
Age		0.98	[0.91-1.05]	0.53
		1		
Sex	Male	0.59	[0.25-1.36]	0.60
	Female	1		
Marital status	Single/ widow	0.44	[0.18-1.09]	0.08
	Married	1		
Longevity in service		1.00	[0.99-1.00]	0.66
Knowledge on healthy skin and infected blood as a risk factor	No	0.53	[0.24-1.16]	0.11
	Yes	1		
Knowledge on minimum number of vaccine dose	No	0.25	[0.10- 0.62]	0.00
	Yes	1		
Screening for HBsAg	Yes	4.09	[1.56- 10.68]	0.00
	No	1		
Attitudes regarding safety of vaccine	No	0.44	[0.175-1.09]	0.08
		1		

## Discussion

Generally, HCW are expected to have adequate knowledge about diseases and other health conditions. Hepatitis B infection is an occupational disease and HCW due to the nature of their job are more at risk. It is in this light that we wanted to assess the influence of knowledge and attitudes on the vaccination status of HCW in the BHD. Out of the 280 HCWs recruited in the study, the majority (35.3%) were nurses followed by laboratory technicians (21.2%). We had fewer specialists and general practitioners 3.9% each, this can be compared to the statistics from the BHD (staffing situation 2010) where 27.8% of HCW vwere nurses next by 18.5% for laboratory technicians (source BHD, 2010). Also more of the respondents 16.9% were from the regional hospital; this is in line with statistics from the BHD, which tells us that the regional hospital has the highest number of HCW (BHD statistics, 2010). Analysing for sex, the majority of respondents were females 180 (64.3%) while a minority were males 100 (35.7%). This reflects the Cameroon demography which shows that there are more females than males in the general population (EDS-MICS, 2011) [17].

The most effective strategy to protect HCW from HBV infection is immunization, which offers effective protection in 90-95% of adults [18]. However, its use among HCW in the developing world is very low. The vaccination coverage against HBV of HCW in the BHD was found to be 13.9% which is comparable to that found in Egypt-15.8% [19]. It is different from 24.5% with a sample size of 49 surgical residents in Yaoundé [19], 40.9% with a sample size of 93 HCWs in Northern Cameroon [20], 12.3% in South West Cameroon of which only 5.5% had received a full dose of vaccine [15], 39% with a sample size of 84 surgeons in 14 sub-Saharan countries [21], 6.2% in Uganda [22]. This wide variability can be explained by differences in the settings, sample size, specialties of HCW used in each study, and economic factors. Comparing our results to the 24.5% in Yaoundé, our value (13.9%) is lower.

Generally, the HCW had sufficient knowledge 62.6% and good attitudes 85.8%. All HCW had sufficient attitudes but for their attitudes regarding the HBsAg test which was not sufficient, only 57.9% of HCW had done this test. Not all specialties had the same knowledge percentage score; specialists had the highest average knowledge of 89% followed by and general practitioners with 86.4%. Pharmacists had the least average knowledge (50.4%) and none of them were vaccinated. This could also be because pharmacists do not come in direct contact with patients hence might not have seen the need for vaccination, they do not know that the virus can last for up to seven days on any surface it comes in contact with and remains infectious. Laboratory technicians, assistant laboratory technicians, nurse assistants, nurses, pharmacists and radiologists did not have sufficient knowledge on the minimum number of doses for HBV vaccination, not all HCW who responded that they were vaccinated had taken a complete dose of the vaccine.

The knowledge of HCW was low in the following; knowledge regarding infected blood and healthy skin as a risk factor, 53.2% responded that it was a risk factor. This can be compared to the 77.6% for those who reported no in a study carried out in Yaoundé. They considered that contact of healthy skin and infected blood was not a risk factor not considering the fact that HBV can last for up to 7days on any surface it comes into contact with and still remain infectious [23].

The knowledge of HCW influenced their vaccination status; this is the same as was reported in a study carried out in Kuwait [24]. Knowledge of HCWs regarding the minimum number of doses for HBV vaccination was 53.8%. HCW who had less knowledge regarding the minimum number of doses for HBV vaccination were less likely to be vaccinated than those who had knowledge (p=0.00; 95% CI: 0.10-0.62; AOR=0.25). This knowledge 53.8% in our study is different from that which was gotten from a study in Yaoundé 69.4% [19] Their result (69.4%) can be compared to 90.9% knowledge for specialists in our study; the difference could be as a result of environment and sample size. As concerns attitudes, their attitudes regarding screening for HBsAg were significantly associated with their vaccination status. Those who had done the HBsAg test were more likely to be vaccinated than those who were not done the test [AOR = 4.09; 90% CI: 1.56-10.68, p=0.00].

There was no significant association between gender and vaccination status of HCW in the BHD. This is different from a study carried out in India where female HCW were more likely to be vaccinated than male HCW [25]. From our study other factors which were not significantly associated with the vaccination status of HCW were; marital status, type of HF (public/private), and location of HF (urban/rural). In addition to the knowledge and attitudes of HCWs, other factors could have influenced the vaccination status of HCWs. This can be seen from the fact that though 90.7% of HCWs knew they were more at risk, 98.6% reported that the virus can be prevented of which 72.6% said the most effective means of prevention was by vaccination, only 13.9% had taken the vaccine. These factors could have been 34.6% for the vaccine is costly, 23.2% for lack of time. These findings were consistent with those of Kesieme EB *et al*, 2011; 1570-1589 [26]. It is different from the findings in Yaoundé where they had 38.5% for lack of time and 23.1% for the high cost of vaccine [19]. This difference could be due to economic factors and differences in population type. Other reasons for low vaccination coverage among HCW were; 13.2% for negligence, 11.4% for vaccine not available, 3.9% for awareness not created in population, 2.5% for vaccine not effective, which can be compared to a study carried out in Nigeria among Obstetricians and midwives where respondents feared contracting HBV from the vaccine [27].

## Conclusion

Knowledge and attitudes of HCW were associated with their vaccination status. Healthcare workers that did not know the minimum number of doses for HBV vaccination were less likely to be vaccinated than those who knew. Also, those who had been screened for HBsAg were more likely to be vaccinated than those who had not been screened. Other factors which could be associated with the vaccination status of HCW were the high cost of the vaccine, lack of time, negligence, and non-availability of the vaccine. The total number of HCW who were correctly vaccinated; was found to 13.9%. There is therefore a need for rapid intervention with actors acting accordingly to increase this vaccination coverage among HCW in the BHD. The research revealed inequities in knowledge; the following specialties of HCW should be educated on the subject of the minimum number of vaccine doses; laboratory technicians, assistant laboratory technicians, nurse assistants, nurses, pharmacists and radiologists. Healthcare workers should be encouraged to go for HBsAg screening and those who are negative should receive a full course of HBV vaccination to avoid chronic HBV infection. There is a need for subsidization of vaccines so that they could be afforded by all HCW. The vaccine should not only be subsidized but also made available and awareness should be created among HCWs through educational talks.

**Limitation of the study:** the sampling method for this study limits us to a non-representative sample size, making it not possible to extrapolate results. Also, the questionnaires were not interviewer-administered (information biased); and as a result, we might not have had quality information from them. Also, some participants responded the next day, this may have led to increased variability in the data and biases. Due to the small sample size, a lot of recording was done, this recoding could have led to the loss of information especially recoding of a specialty of HCW. Also because of the small sample size, we could not use a statistical test to prove that there was a difference in knowledge among the different specialties of HCW recruited in the study.

### What is known about this topic

- Precautions for health care workers to avoid hepatitis B and C virus infection;
- Hepatitis B virus epidemiology, disease burden, treatment, and current emerging prevention and control measures;
- Knowledge, attitudes, and vaccination coverage of healthcare workers regarding occupational vaccinations.

### What this study adds

- Some reasons for low vaccination coverage among HCW were: negligence, no availability of vaccines, lack of awareness, and some vaccines not effective;
- Also, those who had been screened for HBsAg were more likely to be vaccinated than those who had not been screened;
- The research revealed inequities in knowledge; the following specialties of HCW should be educated on the subject of a minimum number of vaccine doses; laboratory technicians, assistant laboratory technicians, nurse assistants, nurses, pharmacist, and radiologists.
